# Acknowledgement of manuscript reviewers 2015

**DOI:** 10.1186/s12944-016-0198-3

**Published:** 2016-03-01

**Authors:** Undurti Das

**Affiliations:** UND Life Sciences, Shaker Heights, USA

## Abstract

The editors of *Lipids in Health and Disease* would like to thank all our reviewers who have contributed to the journal in Volume 14 (2015).

**Aarnoud van der Spoel**

Canada

**Abdelfattah Attallah**

Egypt

**Abir Ben Bacha**

Saudi Arabia

**Agata Stanek**

Poland

**Ahmed El-Sohemy**

Canada

**Ahmed Rebai**

Tunisia

**Ahmet Selçuk Can**

Turkey

**Ake Nilsson**

Sweden

**Akihiro Inazu**

Japan

**Akiyo Matsumoto**

Japan

**Alberico L. Catapano**

Italy

**Alberto Bargossi**

Italy

**Aldo Eynard**

Argentina

**Aldona Dembinska-Kiec**

Poland

**Alessandra Gambero**

Brazil

**Alex Kulik**

United States of America

**Alexandre Carrilho**

Brazil

**Alfonso Bellia**

Italy

**Ali Tiss**

Kuwait

**Allan Zhao**

China

**Alvaro Cerda**

Brazil

**Amanda Baviera**

Brazil

**Amedeo Lonardo**

Italy

**Amirhosein Khoshi**

Islamic Republic of Iran

**Amitabha Chattopadhyay**

India

**Ammara Yasmeen**

Sri Lanka

**Ana Claudia Losinskas Hachul**

Brazil

**Ancha Baranova**

United States of America

**Anders Gammelmark**

Denmark

**Andrew Thomas**

United Kingdom

**Anetta Undas**

Poland

**Angelo Baldassare Cefalu**

Italy

**Anilkumar Gopalakrishnapillai**

India

**Annamaria Morelli**

Italy

**Anne Grete Semb**

Norway

**Annemarie Gagnon**

Canada

**Anthony Wierzbicki**

United Kingdom

**Arrigo Fg Cicero**

Italy

**Arulmurugan Mani**

India

**Arvind Shah**

United States of America

**Ashraf Gorgey**

United States of America

**Asley Snider**

United States of America

**Ayako Tsuchiya**

Japan

**Bahareh Asadishad**

Canada

**Barbara Meyer**

Australia

**Bashir Jarrar**

Jordan

**Belur Lokesh**

India

**Bill Lands**

United States of America

**Brian Tomlinson**

Hong Kong

**Bruno M Fonseca**

Portugal

**C Richter**

United States of America

**Camille Balarini**

Brazil

**Carlos O Mendivil**

United States of America

**Carlos Santos-Gallego**

United States of America

**Caroline Pettersen**

Norway

**Caroline Schmidt**

Sweden

**Catherine Calzada**

France

**Catherine Mounier**

Canada

**Charlotte Jacobsen**

Denmark

**Christian Diot**

France

**Christina Christoffersen**

Denmark

**Christine Christiansen**

Denmark

**Christos Pitsavos**

Guam

**Cinzia Parolini**

Italy

**Claudia Cardoso**

Brazil

**Claudio Orsatti**

Brazil

**Costantinos Demopoulos**

Greece

**Cresio Alves**

Brazil

**Dagmar Kratky**

Austria

**Daisuke Ekuni**

Japan

**Dalia Hamdy**

Egypt

**Damjana Rozman**

Slovenia

**Daniel Gaudet**

Canada

**Daniel Gelain**

Brazil

**Danijela Gasevic**

Canada

**Danik Martirosyan**

United States of America

**Danxia Yu**

United States of America

**Dariusz Mikulski**

Poland

**David Dyck**

Canada

**David Freedman**

United States of America

**Debora Estadella**

Brazil

**Demosthenes Panagiotakos**

Greece

**Denise Carmona Cara**

Brazil

**Diego F Calvisi**

Germany

**Dimitris Tousoulis**

Greece

**Dirk Blom**

South Africa

**Dongyin Guan**

United States of America

**Eder CR Quintao**

Brazil

**Ederlan Ferreira**

Brazil

**Edward Janus**

Australia

**Efstathios Koulouridis**

Greece

**Elisabetta Albi**

Italy

**Elisardo C Vasquez**

Brazil

**Elizabethe Esteves**

Brazil

**Elzbieta Kimak**

Poland

**Emma Allott**

United States of America

**Enzo Manzato**

Italy

**Erick de Oliveira**

Brazil

**Erik Karlsson**

United States of America

**F Mccarthy**

Ireland

**F Paula**

Brazil

**Fabio Lira**

Brazil

**Fareed Arthur**

Ghana

**Fenghong Huang**

China

**Fernando Civeira**

Spain

**Fiorella Biasi**

Italy

**Fook Tim Chew**

Singapore

**Francisc Vasile Dulf**

Romania

**Frederic Capel**

France

**Frederic Preitner**

Switzerland

**Frederico Neves**

Brazil

**Fredric Kraemer**

United States of America

**Fumihiko Kamezaki**

Japan

**G Pueschel**

Germany

**Gabriele Riccardi**

Italy

**Gaojun Cai**

China

**George Kalliolias**

United States of America

**Gianfranca Carta**

Italy

**Giuliana Fortunato**

Italy

**Gleisson Brito**

Brazil

**Gordon Doig**

Australia

**Gösta Eggertsen**

Sweden

**Gregor Berger**

Switzerland

**Guiyuan Ji**

China

**Gunther Marsche**

Austria

**Gustavo Duarte Pimentel**

Brazil

**Guy Rousseau**

Canada

**Hamidreza Goodarzynejad**

Iran

**Harald Osmundsen**

Norway

**Helena Gylling**

Finland

**Helene Cote**

Canada

**Hiroyuki Itabe**

Japan

**Hubert Scharnagl**

Austria

**Ian Godsland**

United Kingdom

**Ian Newhouse**

Canada

**Ichiro Sakuma**

Japan

**Ilias Giannenas**

Greece

**Irena King**

United States of America

**Isabelle Leclercq**

Belgium

**Isela Parra-Rojas**

Mexico

**Ivana Celap**

Croatia

**Iwona Rudkowska**

Canada

**J Laparra**

Spain

**Jan Kovar**

Czech Republic

**Jan Philipp Schuchardt**

Germany

**Jane Chang**

Taiwan

**Jean-Marc Lavoie**

Canada

**Jeffrey Horowitz**

United States of America

**Jeffrey Hurst**

United States of America

**Jenifer Sassarini**

United Kingdom

**Jeremy Schaefer**

United States of America

**Jerzy Beltowski**

Poland

**Jette Young**

Denmark

**Jiajun Zhao**

China

**Jill Parnell**

Canada

**Jing-Hsien Chen**

Taiwan

**Joachim De Klerk Uys**

United States of America

**Joan Carles Escola-Gil**

Spain

**John Flack**

United States of America

**John Stafford**

United States of America

**Jonathan Alistair Cook**

United Kingdom

**Jonathan Cedernaes**

Sweden

**Jones Graceli**

Brazil

**Jorge Joven**

Spain

**Jorie Versmissen**

Netherlands

**Jorma Viikari**

Finland

**Jos Brouwers**

Netherlands

**José Rosa Neto**

Brazil

**Juan Diaz-Zagoya**

Mexico

**Jude Uzoma Ohaeri**

Nigeria

**Julia Goedecke**

South Africa

**Julie Chowen**

Spain

**Jun Hata**

Japan

**Kalypso Karastergiou**

United States of America

**Kamal Amin**

Egypt

**Kamalan Jeevaratnam**

Malaysia

**Karasawa Ken**

Japan

**Katerina Skenderi**

Greece

**Kathryn Meier**

United States of America

**Kazuhiko Kotani**

Japan

**Kazuhisa Tsukamoto**

Japan

**Kazutoshi Murakami**

Japan

**Keizo Kasono**

Japan

**Ken Ebihara**

Japan

**Kevin Fritsche**

United States of America

**Khaled Hamden**

Tunisia

**Koji Nagao**

Japan

**Konstantinos Tziomalos**

Greece

**Krishanu Sengupta**

India

**Krishnapura Srinivasan**

India

**Kristie Bridges**

United States of America

**Lars Berglund**

United States of America

**Laszlo Boros**

Hungary

**Laszlo G. Puskas**

Hungary

**Laszlo Puskas**

Hungary

**Leda Vieira**

Brazil

**Lenka Taylor**

Germany

**Lila Oyama**

Brazil

**Lilian Thompson**

Canada

**Livia Pisciotta**

Italy

**Loo Keat Wei**

Malaysia

**Loredan Stefan Niculescu**

Romania

**Lucia Terzuoli**

Italy

**Luis Lopez**

United States of America

**Luiz Claudio Fernandes**

Brazil

**Luiz Guilherme Kraemer-Aguiar**

Brazil

**M Andreoli**

Argentina

**M Elizabeth Sublette**

United States of America

**Maciej Banach**

Poland

**Madiha Dhibi**

Tunisia

**Makoto Kurano**

Japan

**Makoto Makishima**

Japan

**Mala Ganesan**

India

**Malika Bouchenak**

Algeria

**Manfredi Rizzo**

Italy

**Mani Mirfeizi**

Islamic Republic of Iran

**Manuel Coelho-e-Silva**

Portugal

**Marco Matteo Ciccone**

Italy

**Maria Alonso**

Spain

**Maria Fernanda Cury-Boaventura**

Brazil

**Maria Luz Fernandez**

United States of America

**Maria Maraki**

Greece

**Maria Stepanova**

United States of America

**Mariann Harangi**

Hungary

**Maricela Rodriguez-Cruz**

Mexico

**Marijana Todorcevic**

United Kingdom

**Marin Carmen**

Spain

**Mario Hiroyuki Hirata**

Brazil

**Mary Harris**

Japan

**Meenakshi Sharma**

India

**MeherVani Chaduvula**

India

**Mengyang Liu**

China

**Michael Davidson**

United States of America

**Michael Rene Skjelbo Nielsen**

Denmark

**Michael Ullian**

United States of America

**Michel P Hermans**

Belgium

**Miki Igarashi**

United States of America

**Min Lou**

China

**Minoru Okubo**

Japan

**Miquel Mulero**

Spain

**Mohammadreza Vafa**

Iran

**Muhammad Sajid Arshad**

Sri Lanka

**Mustafa Kemal Balci**

Turkey

**Mustafa Saygin**

Turkey

**Mutay Aslan**

Turkey

**Mykola Khalangot**

Ukraine

**Naoki Hashimoto**

Japan

**Naoki Sakane**

Japan

**Nazia Begum**

India

**Nelo Zanchi**

Brazil

**Nicolo Merendin**

Italy

**Niels Nijstad**

Netherlands

**Niki Katsiki**

Italy

**Nikolaos Andrikopoulos**

Greece

**Nipon Chattipakorn**

Thailand

**Nurten Aksoy**

Turkey

**Odunayo Azeez**

South Africa

**Olufunmilayo Adeleye**

Nigeria

**Oscar Perez-Mendez**

Mayotte

**Patricia Lisboa**

Brazil

**Paul Grippo**

United States of America

**Paul Holvoet**

Belgium

**Paul Hulshof**

Netherlands

**Paulo Matafome**

Portugal

**Peter Kisfali**

Hungary

**Peter Sabaka**

Slovakia

**Peter Toth**

United States of America

**Philip Chilibeck**

Canada

**Philippe Delerive**

France

**Piegari Mariana**

Argentina

**Poonamjot Deol**

United States of America

**Priscila Maranhão**

Brazil

**Qi Mi**

United States of America

**Qilin Gu**

United States of America

**Qing Duan**

United States of America

**Rashida Parveen**

Pakistan

**Raul Barrera Rodriguez**

Mexico

**Rejean Couture**

Canada

**Rob Weijers**

Netherlands

**Robert Elkin**

United States of America

**Robert Hegele**

Canada

**Robin Clugston**

United States of America

**Rodrigo Neves**

Brazil

**Roman Alberty**

Slovakia

**Rong Mu**

United States of America

**Rudolf Poledne**

Czech Republic

**Sadahiko Iwamoto**

Japan

**Saeid Ghavami**

Canada

**Saikat Dewanjee**

Italy

**Sandro Hirabara**

Brazil

**Sasa Frank**

Austria

**Satoshi Fujii**

Japan

**Scott Blackwell**

United Kingdom

**Sebastiao Freitas de Medeiros**

Brazil

**Sekhar Kambakam**

United States of America

**Seth Martin**

United States of America

**Sevda Tanrikulukucuk**

Turkey

**Shawn Ritchie**

Canada

**Shelley Tworoger**

United States of America

**Shengrong Shen**

China

**Shiming Li**

United States of America

**Shlomo Vinker**

Israel

**Sibel Silici**

Turkey

**Signe Borgquist**

Sweden

**Simon Dyall**

United States of America

**Smaragdi Antonopoulou**

Greece

**Stefan Lorkowski**

Germany

**Steven Krilis**

Australia

**Stine Marie Ulven**

Norway

**Sungjin Chung**

Republic of Korea

**Susanne Dyby**

United States of America

**Sushant Bhatnagar**

United States of America

**Suzana Jerez**

Argentina

**Suzanne Al-Bustan**

Kuwait

**Suzanne Jackowski**

United States of America

**Tahira Fatima**

Canada

**Takahiro Fujino**

Japan

**Takeshi Sakurai**

Japan

**Taku Inoue**

Japan

**Takuya Kimura**

Japan

**Tao Huang**

United States of America

**Tatiana Emanuelli**

Brazil

**Tatjana Ábel**

Hungary

**Tatjana Stojakovic**

Austria

**Teodoro Marotta**

Italy

**Theodoros Kelesidis**

United States of America

**Thereza Bargut**

Brazil

**Tiago De Melo**

Brazil

**Timothy Hla**

United States of America

**Tom Kovala**

Canada

**Toru Hosoi**

Japan

**Tsuyoshi Nozue**

Japan

**Ujendra Kumar**

Canada

**Undurti Das**

United States of America

**Uwe Tietge**

Netherlands

**Vasilios Athyros**

Greece

**Vera Silveira**

Brazil

**VGM Naidu**

India

**Vincenzo Tufarelli**

Italy

**Vito Laudadio**

Italy

**Vladimir Palicka**

Czech Republic

**W Harris**

United States of America

**Welma Stonehouse**

Australia

**Welma Stonehouse**

New Zealand

**Wensheng Pan**

China

**Wilfried Le Goff**

France

**Wolfgang Kratzer**

Germany

**Xiangbo Ruan**

United States of America

**Xiaobo Lin**

United States of America

**Xiaodong Zhang**

United States of America

**Xueqiu Jian**

United States of America

**Yesim Oztas**

Turkey

**Yongjie Ma**

United States of America

**Yoshifusa Aizawa**

Japan

**Yoshimi Kishimoto**

Japan

**Yoshio Fujioka**

Japan

**Yoshitaka Hayashi**

Japan

**You-Lin Tain**

Taiwan

**Youssef Attia**

Egypt

**Yuji Shimizu**

Japan

**Yuming Wang**

China

**Yutao Zhan**

China

**Zeqin Lian**

China

**Zhirajr Mokini**

Italy

**Zhong Chen**

China

**Zhongkai Zhou**

China

**Zhongqiu Xie**

United States of America

